# The Assembly of HTLV-1—How Does It Differ from HIV-1?

**DOI:** 10.3390/v16101528

**Published:** 2024-09-27

**Authors:** Dominik Herrmann, Shuyu Meng, Huixin Yang, Louis M. Mansky, Jamil S. Saad

**Affiliations:** 1 Department of Microbiology, University of Alabama at Birmingham, Birmingham, AL 35294, USA; dominik.herrmann@regeneron.com; 2 Institute for Molecular Virology, University of Minnesota–Twin Cities, Minneapolis, MN 55455, USA; meng0137@umn.edu (S.M.); yang5928@umn.edu (H.Y.); mansky@umn.edu (L.M.M.); 3 Molecular Pharmacology and Therapeutics Graduate Program, University of Minnesota–Twin Cities, Minneapolis, MN 55455, USA; 4 Department of Diagnostic and Biological Sciences, University of Minnesota–Twin Cities, Minneapolis, MN 55455, USA; 5 Masonic Cancer Center, University of Minnesota–Twin Cities, Minneapolis, MN 55455, USA; 6 Department of Biochemistry, Molecular Biology and Biophysics, University of Minnesota–Twin Cities, Minneapolis, MN 55455, USA

**Keywords:** human T-cell leukemia virus type 1 (HTLV-1), human immunodeficiency virus type 1 (HIV-1), Gag polyprotein, matrix (MA), capsid (CA), plasma membrane (PM), phosphatidylinositol 4,5-bisphosphate (PI(4,5)P_2_)

## Abstract

Retroviral assembly is a highly coordinated step in the replication cycle. The process is initiated when the newly synthesized Gag and Gag-Pol polyproteins are directed to the inner leaflet of the plasma membrane (PM), where they facilitate the budding and release of immature viral particles. Extensive research over the years has provided crucial insights into the molecular determinants of this assembly step. It is established that Gag targeting and binding to the PM is mediated by interactions of the matrix (MA) domain and acidic phospholipids such as phosphatidylinositol 4,5-bisphosphate (PI(4,5)P_2_). This binding event, along with binding to viral RNA, initiates oligomerization of Gag on the PM, a process mediated by the capsid (CA) domain. Much of the previous studies have focused on human immunodeficiency virus type 1 (HIV-1). Although the general steps of retroviral replication are consistent across different retroviruses, comparative studies revealed notable differences in the structure and function of viral components. In this review, we present recent findings on the assembly mechanisms of Human T-cell leukemia virus type 1 and highlight key differences from HIV-1, focusing particularly on the molecular determinants of Gag–PM interactions and CA assembly.

## 1. Discovery of HTLV-1

Prior to their discovery in the early 1980s, the possible existence of human retroviruses was highly controversial. Many factors, including multiple reports of “discoveries” of human retroviruses that turned out to be cross-contaminations with animal retroviruses, contributed to the consensus that human retroviruses did not exist. Two important technical advances in the early 1970s specifically fueled the discovery of the first human retrovirus, human T-cell leukemia virus type 1 (HTLV-1) [1,2]. First, a novel T-cell mitogenic factor, later called interleukin-2 (IL-2), was characterized which allowed the cultivation of primary blood cells. Second, a sensitive reverse transcriptase (RT) assay was developed, distinguishing RT from all other known cellular polymerases [3,4]. These technical advances led to evidence of the presence of a human retrovirus (i.e., HTLV-1) [5,6], which paved the way for the subsequent discovery of human immunodeficiency virus type 1 (HIV-1) as the etiological agent of acquired immunodeficiency disease syndrome (AIDS). HTLV-1 is classified in the *deltaretrovirus* genus of the subfamily *orthoretrovirinae.*

## 2. Epidemiology

It has been difficult to accurately estimate the number of HTLV-1 carriers globally as prevalence studies are lacking to allow for such determinations to be made. Some estimates of the total number of people infected with HTLV-1 have ranged from 5 to 10 million, although the number is arguably higher due to the lack of adequate data (World Health Organization [7]). There are several factors complicating such studies: (1) HTLV-1 is known to be highly localized in certain clusters or foci of endemicity and it is likely that existing clusters are not identified; (2) reliable epidemiological data are not available from many highly populated countries in the world, including China, India, and East and Northwest Africa; and (3) epidemiological data are obtained through screening of volunteers (e.g., blood donors, pregnant women, or hospitalized patients), and therefore may not be entirely representative of the general population, introducing a selection bias. Despite these challenges in accurately determining total HTLV-1 infection rates worldwide, areas of high endemicity are known to include South America, Caribbean islands, sub-Saharan Africa, Japan, and central Australia, whereas seroprevalence of HTLV-1 within the United States appears to be low (5 cases/100,000 among first-time blood donors) [8]. A previous study has shown that HTLV-1 infection increases with age, in all areas with high HTLV-1 prevalence, which is likely due to the accumulation of sexual contacts and in general transmission events over time [8]. Taken together, due to the lack of reliable epidemiological data, the actual prevalence of HTLV-1 carriers is likely to be much higher than most current estimates.

There are three major ways HTLV-1 can be transmitted from one person to another. First, vertical mother-to-child transmission via prolonged breastfeeding (>6 months) increases the likelihood of up to 25% of virus transmission events [9,10,11,12,13,14]. Second, horizontal transmission via blood transfusions from HTLV-1 seropositive donors represents a major risk, with up to 63% of recipients acquiring HTLV-1 infection. Intravenous drug use also presents a major risk of blood-borne horizontal transmission [15]. Third, the virus can be transmitted sexually, more frequently from men to women, which helps explain why seroprevalence increases with age, specifically in women [16,17,18,19,20].

HTLV-1 is expected to remain a persistent global health issue. Although HTLV-1 is less known compared to other viruses like HIV-1, it remains a significant health concern due to its potential to cause serious diseases (see below). Increased globalization and migration could lead to changes in the distribution of HTLV-1. As people move between regions with different prevalence rates, there is potential for new outbreaks or the spread of the virus to areas previously less affected. Studying HTLV-1 is crucial for informing public health strategies and policies, especially in regions with high prevalence. Effective screening programs, prevention strategies, and educational initiatives are needed to manage and reduce the burden of HTLV-1-related diseases.

## 3. Pathology

Chronic HTLV-1 infection is associated with two major diseases, adult T-cell leukemia/lymphoma (ATLL) and HTLV-1-associated myelopathy/tropical spastic paraparesis (HAM/TSP) [5,6,21,22,23,24]. ATLL is a mature T-lymphoid malignancy of post-thymic pleomorphic activated T lymphocytes. It is classified into four clinical forms (i.e., acute, chronic, smouldering, and lymphomatous) based on leukemic manifestation in the blood, with organ involvement, and by lactate dehydrogenase and serum calcium levels [25]. Acute ATLL is the most common malignancy, with ~65% of patients presenting with this aggressive, fast-growing type of ATLL. Hematopathological features include a raised white blood cell count along with atypical “flower cells”, named for their petal-shaped, polylobated nuclei. The lifetime risk of developing ATLL is higher in men (4–6%) than in women (2.6%), and is associated with a high proviral load, age, and based upon certain genetic predispositions [22,23,26,27,28]. Current treatment options include antiviral therapy using zidovudine plus interferon-alfa (AZT/IFN), multi-agent chemotherapy, or allogeneic hematopoietic stem cell transplantation (allo-HSCT) [29]. The overall prognosis of acute ATLL remains poor, with a mean survival time of less than one year using traditional treatment methods [25]. However, higher survival rates have been reported for individuals receiving allo-HSCT [30].

HAM/TSP, also described as “chronic progressive parainfectious myelitis”, is pathologically characterized as a progressive inflammation and subsequent degeneration of white and gray matter in the spinal cord [31]. This degeneration results in a variety of neurological symptoms, including spasticity and weakness of the lower extremities. Disease onset has commonly been observed within the fourth and fifth decade of life, with a higher prevalence in women than in men (i.e., 3:1 ratio). Neurological symptoms have been observed to develop in some individuals between two months and three years following a blood transfusion contaminated with HTLV-1-infected T-cells. This strong correlation supports the hypothesis that HTLV-1 transmission through such transfusions is a viable model, suggesting that HAM/TSP can develop relatively quickly after infection [31]. Up to 4% of HTLV-1-infected individuals eventually develop HAM/TSP [32]; however, the exact mechanism of disease development is unclear. It is speculated that chronic activation of HTLV-1-specific immune responses may be related to higher expression levels of HTLV-1 antigens in individuals developing HAM/TSP [32]. Additionally, a recent study demonstrated that a specific population of HTLV-1 specific CD8^+^ cytotoxic T-cells expressing a unique T-cell receptor can trigger an excessive immune response against HTLV-1 infected cells infiltrating the cerebrospinal fluid in HAM/TSP patients [33]. Furthermore, a high proviral load due to the insertion of the viral genome in a highly transcriptionally active region of the genome was associated with HAM/TSP [34].

## 4. Routes of Transmission

The primary target of HTLV-1 in vivo is CD4^+^ T-cells [35,36,37]; however, infection of CD8^+^ T-cells, dendritic cells, and monocytes has also been shown to a lesser extent [38,39,40]. In contrast to HIV-1, infection via free HTLV-1 particles is not efficient. In fact, plasma derived from HTLV-1-infected patients is not thought to be infectious at all, either due to a lack of free virions in the plasma, or virions contained in the plasma not being infectious [41]. Instead, direct cell-to-cell contact is the main mode of transmission for HTLV-1, both in vitro and in vivo [42,43,44,45]. Two mechanisms of cell-to-cell transmission have been proposed: (1) Virions assemble at the virological synapse, a tight junction between the infected and the uninfected host cell, and bud directly into the synaptic cleft, or (2) a viral biofilm is transferred on the cell surface at virological synapses [46]. Both routes provide enhanced protection from immune recognition during transmission when compared to infection via free virus particles [47,48].

During the cell-to-cell transmission of HTLV-1, the cytoskeleton of the infected cell is remodeled leading to polarization of the microtubule organizing center (MTOC) towards the cell-cell junction. Subsequently, Gag and Env proteins as well as viral RNA localize to the cell-cell junction [49]. It has been shown that the inhibition of actin and tubulin polymerization inhibited MTOC organization and HTLV-1 infectivity by more than 95%, indicating that interactions of viral proteins required for assembly with cellular proteins associated with the cytoskeleton are essential for viral infectivity [49,50]. However, the molecular details of how Env and Gag localize at cell-to-cell junctions remain to be understood.

## 5. Attachment, Fusion, and Entry

**Figure 1:**
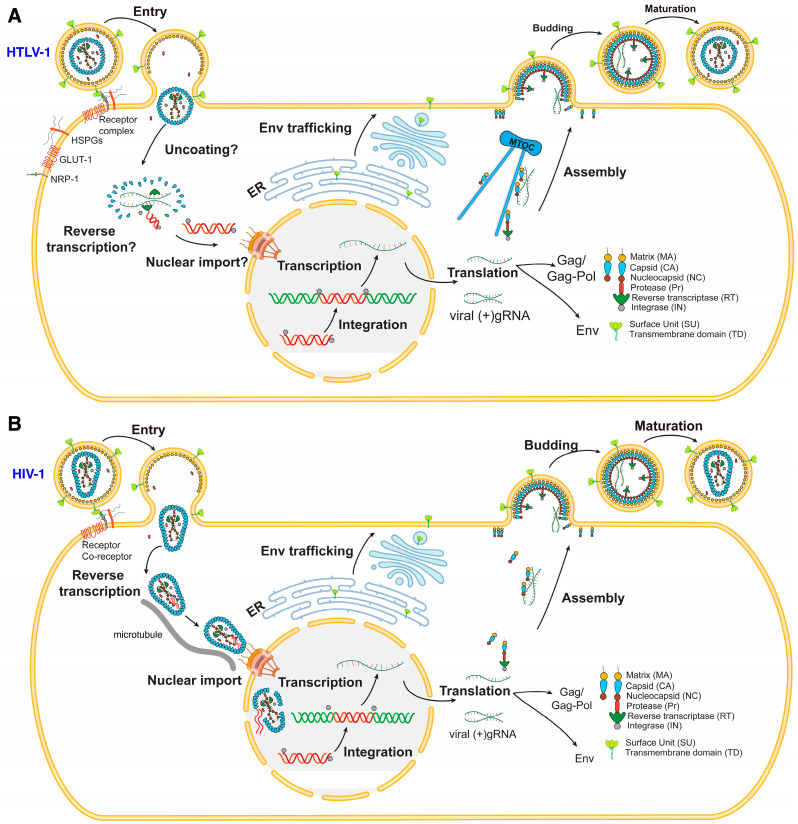
HTLV-1 and HTLV-1 replication cycles. (**A**) Mature HTLV-1 virion attaches to the host cell receptor complex containing NRP-1, GLUT-1, and HSPGs. The mechanisms of reverse transcription and uncoating have long been thought to occur in the cytoplasm but recent advances on the mechanisms of HIV-1 reverse transcription and uncoating (below) raised similar questions about other retroviruses, including HTLV-1. Subsequent nuclear import and integration into the host genome yields the provirus. Transcription and translation produce Gag, Gag-Pol, Env, accessory proteins, and viral gRNA. Gag is trafficked to the PM for assembly via the MTOC, while Env is post-translationally processed and trafficked to the cell surface through the ER and Golgi apparatus. Virus assembly and maturation yield a new, infectious virus. (**B**) Mature HIV-1 virion attaches to the host CD4 receptor and co-receptors (CCR5 or CXCR4). The virus core is then transported to the nucleus via microtubules, a process that appears to be accompanied by reverse transcription. Recent studies indicated that CA core uncoating occurs in the nucleus near the integration sites. Transcription and translation produce Gag, Gag-Pol, Env, accessory proteins, and viral gRNA. Gag is then trafficked to the PM for assembly, while Env is post-translationally processed and trafficked to the cell surface through the ER and Golgi apparatus. Virus assembly and maturation yield a new, infectious virus.

Attachment of HTLV-1 to the host cell is facilitated by the binding of the surface subunit (SU) gp46 to a receptor complex on the cell surface, which includes glucose transporter 1 (GLUT1) [36,51], heparan sulfate proteoglycans (HSPGs) [35,52], and neuropilin-1 (NRP-1) [53,54,55] (Figure 1A). A possible sequence of events for cell entry involves the virus first interacting with HSPGs, then with NRP-1, followed by GLUT1, ultimately leading to membrane fusion. However, the precise details of these interactions remain unclear. Despite these receptors being present in various cell types, HTLV-1 is predominantly found in CD4+ T-cells. This preferential detection may be attributed to increased proliferation of CD4+ T-cells following HTLV-1 infection [56]. After the viral membrane has fused with the cellular membrane, the viral capsid (CA) shell, containing two copies of genomic RNA (gRNA), enters the cytoplasm. This marks the beginning of the HTLV-1 replication cycle, which involves reverse transcription of the viral RNA genome. The specifics of post-entry processes, including uncoating, reverse transcription, and nuclear import, are not well understood.

Conversely, over the past three decades, extensive research on HIV-1 attachment, membrane fusion, and entry has resulted in a detailed understanding of these mechanisms (reviewed in [57]). Briefly, the Env protein which is initially produced as a precursor known as gp160, forms a trimer structure and then undergoes cleavage by a host furin-like protease, resulting in two noncovalently associated fragments: gp120, involved in CD4 receptor binding, and gp41, which is responsible for fusion [57]. The mature viral spike is composed of three copies of each fragment (gp120/gp41)_3_. Virus attachment is initiated when gp120 binds to CD4, triggering conformational changes and allowing it to bind to a coreceptor (CCR5 or CXCR4), leading to refolding of gp41 (Figure 1B) [58]. The cleavage between gp120 and gp41 leaves the protein in a metastable state relative to its postfusion conformation. Upon receptor binding by gp120, the N-terminal fusion peptide (FP) of gp41 translocates and inserts into the target cell membrane. This is followed by the refolding of gp41 into a hairpin conformation, forming a six-helix bundle known as the postfusion conformation. This arrangement brings the FP and transmembrane segments to one end of the molecule, facilitating the fusion of the viral and target cell membranes. This process involves the formation of a hemifusion stalk and subsequent fusion pore, leading to the entry of the viral CA into the target cell.

## 6. Uncoating, Reverse Transcription, and Integration

Whereas the timing, sequence, and mechanisms of uncoating and reverse transcription have been extensively studied for HIV-1, similar studies have yet to be performed on HTLV-1. Recent studies provided compelling evidence that HIV-1 uncoats in the nucleus, near the integration site (Figure 1B) [59,60,61,62,63,64,65]. It has been also shown that the efficient uncoating of nuclear HIV-1 cores requires the synthesis of a double-stranded DNA genome > 3.5 kb and that the efficiency of core uncoating correlates with genome size [65]. It is unclear whether reverse transcription and uncoating of HTLV-1 are linked, or whether the CA core can enter the nucleus prior to uncoating, as is the case with HIV-1 (Figure 1B) [66]. Additionally, the initial trigger for reverse transcription in HTLV-1 is not well defined, though the viral RT begins converting the RNA genome into complementary DNA (cDNA) soon after entering the host cytoplasm, forming the RT complex (RTC). As reverse transcription progresses, CA molecules are likely lost from the RTC. For HIV-1, CA is crucial for reverse transcription and nuclear entry [59,60,61,62,63]; however, the role of CA in these processes for HTLV-1 is not well understood. It is also unclear whether reverse transcription in HTLV-1 is completed in the cytoplasm or the nucleus.

Translocation into the nucleus is required for the integration of the viral DNA into the host genome. The integrase (IN) enzyme plays a crucial role in this process by binding to the long terminal repeats (LTRs) of the proviral DNA to form a DNA/IN complex known as the intasome [67,68,69]. IN then processes the 3′ ends of the viral DNA by removing several nucleotides, creating the reactive 3′-OH groups necessary for the enzymatic reaction of integration. The intasome then interacts with the host DNA’s phosphate backbone, leading to the irreversible insertion of the proviral DNA. This proviral DNA is subsequently replicated with each cell division [70,71,72,73]. In vivo, HTLV-1 proviral DNA integrates into transcriptionally active regions and areas with high gene density [74]. However, in vitro studies with HeLa cells suggest that HTLV-1 does not exhibit a preferred integration site [75], similar to the integration patterns in avian sarcoma virus (ASV). The integration site preferences for different retroviruses are likely influenced by structural similarities in the IN enzyme, interactions with chromosomal proteins, or variations in interactions with host factors [67,75,76].

## 7. HTLV-1 Genome

**Figure 2:**
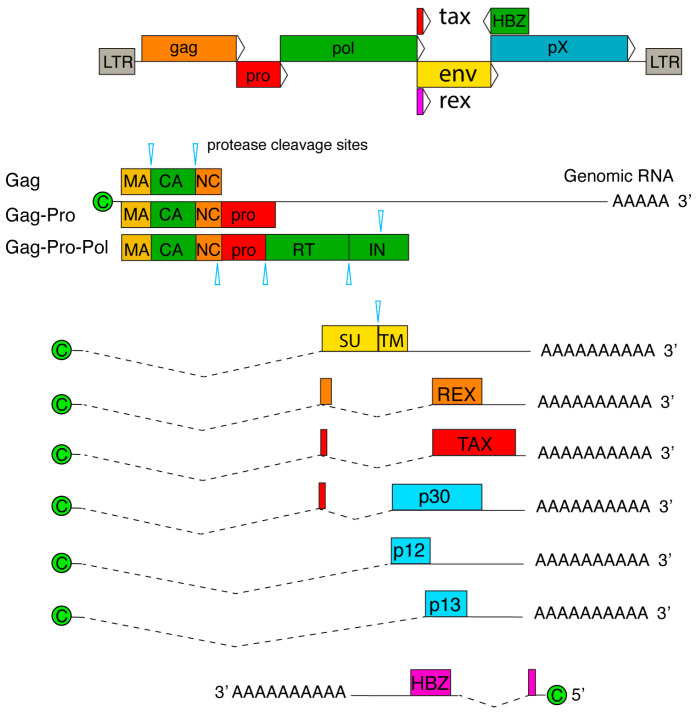
HTLV-1 genome and RNA transcripts. Genome encodes for Gag, Pro, Pol, Env, Tax, Rex, and pX genes. pX region contains genes of Rex, Tax, p30, p12, p13, and HBZ (antisense transcript). mRNA transcripts are 5′-capped and 3′-polyadenylated. Alternative splicing yields mRNA for Env, Tax, Rex, p12, p13, p30, and HBZ.

The HTLV-1 genome is 9.1 kb long, flanked by 5′ and 3′ LTRs. The unspliced, full-length gRNA serves as the viral genome and as a template for Gag, Pro, and Pol (Figure 2). Regulatory and accessory genes are located downstream of the envelope (Env) gene and are denoted as the pX region. The pX region contains multiple, overlapping open reading frames (ORFs), encoding for viral factors such as transactivator protein (Tax), Rex, p12, p13, and p30, which are transcribed via leaky scanning and differential splicing of the viral RNA [24,77]. Tax, a 40-kDa protein, interacts with numerous host proteins to modulate key signaling pathways, including the NF-κB pathway, which is crucial for the survival, proliferation, and transformation of HTLV-1-infected T-cells [24]. Tax alone is capable of immortalizing human T-cells in vitro, highlighting its role as a major oncogenic factor. It promotes cellular transformation by inhibiting DNA repair mechanisms, inducing DNA damage, and inactivating the tumor suppressor p53 [78,79,80,81,82,83]. Additionally, Tax binds to the Tax responsive element I (TRE-1) in the HTLV-1 LTR, which consists of three 21-base pair repeats [79]. This interaction facilitates the recruitment of host factors such as cyclic AMP response element-binding proteins (CREB) and CREB-binding proteins (CBP/p300), which are necessary for the transactivation of the viral promoter and initiation of transcription [84,85]. Rex, a 27-kDa RNA-binding protein, is crucial for viral replication as it stabilizes and exports unspliced gag/pol mRNA and singly spliced env mRNA from the nucleus [86,87,88,89]. Interestingly, the HTLV-1 genome also encodes an antisense basic leucine zipper factor (HBZ) which acts as an agonist to Tax [90,91]. HBZ RNA promotes T-cell proliferation, while HBZ protein suppresses Tax-mediated viral transcription through the 5′ LTR, indicating its dual role in different molecular forms [92,93]. The accessory proteins p12, p13, and p30 are important for viral infectivity in vivo but appear to be dispensable in vitro [24,94].

## 8. Genome Packaging

During the late stage of the HTLV-1 replication cycle, the full-length, unspliced gRNA serves as a template for the translation of Gag, Gag-Pro, and Gag-Pol proteins as well as genome packaging into assembling virions. The Gag protein is composed of three major domains, matrix (MA), CA, and nucleocapsid (NC). Gag and Pol are separated by Pro, which overlaps both at the 3′-region of Gag and the 5′-region of Pol [88]. Ribosomal frameshifts within Gag and Pro result in relatively lower expression of Gag-Pro and Gag-Pro-Pol, generating an estimated ratio of Gag to Gag-Pro/Gag-Pro-Pol of 20:1. This ratio is conserved across retroviruses [95]; altering this ratio has been shown to impact RNA binding and significantly reduce viral infectivity in HIV-1 [95]. HTLV-1 particles are assembled with two unspliced copies of gRNA, selected from a pool of viral and cellular RNAs [96]. Selective genome packaging is mediated by specific interactions between the NC domain of Gag and the gRNA packaging signal (Ψ), located in the 5′-untranslated region (5′-UTR) [97]. The 5′-UTR also plays a role in regulating gRNA dimerization, initiating reverse transcription, and translation through interactions with cellular factors [97,98]. Recent studies have shown that the MA domain of HTLV-1, but not the NC domain, binds short hairpin RNAs derived from the putative Ψ site [98]. The primer-binding site and a region within the Ψ site form stable hairpins that interact with MA. Additionally, besides the known palindromic dimerization initiation site (DIS), a second DIS in HTLV-1 gRNA has been discovered, with both palindromic sequences specifically binding to the NC domain [98]. Unlike HIV-1, where gRNA dimerization is crucial for efficient RNA packaging ([99,100] and references therein), gRNA dimerization does not seem to be necessary for effective RNA packaging in HTLV-1 [98].

## 9. Gag Oligomerization

Retroviral Gag oligomerization, the process by which Gag proteins assemble into higher-order structures known as lattices, is crucial for the proper formation of immature viral particles. Disruption of Gag oligomerization can result in the production of abnormal or non-infectious virions, significantly impacting viral replication and pathogenicity [101,102]. For HIV-1, Gag proteins are initially present as monomers and low-order multimers (e.g., dimers) in the cytoplasm before they are targeted to the plasma membrane (PM). Higher-order Gag multimers are formed mainly at the PM [103]. Gag interactions with viral RNA occur in the cytoplasm of the infected cell and are independent of Gag’s ability to localize to the PM. Nucleic acids also promote the efficient assembly of Gag in vitro [104,105,106]. Comparative studies of subcellular localization revealed that HTLV-1 Gag localizes on the PM at low cytoplasmic concentrations as a monomer, while HIV-1 Gag forms higher-order oligomers in the cytoplasm before membrane binding [107]. At the PM, this results in the formation of Gag clusters that can be visualized as Gag puncta using fluorescent tagging techniques [108]. Leveraging photoconvertible fluorescent proteins and total internal reflection fluorescence microscopy, studies have shown that additional HTLV-1 Gag molecules were recruited to Gag puncta primarily from the PM, whereas HIV-1 Gag puncta biogenesis occurred by recruitment of cytoplasmic Gag molecules [108].

In addition to NC–RNA interactions, Gag multimerization on the PM is primarily driven by the CA domain. Advances in cryo-electron microscopy (cryo-EM) and cryo-electron tomography (cryo-ET) techniques have provided high-resolution structural insights into immature HIV-1 CA, revealing a hexameric arrangement [109,110,111]. Cryo-ET studies of immature HIV-1 particles revealed that Gag hexamers are formed by subsequent additions of Gag dimers, indicating that Gag dimers form the basic building block for assembling the Gag hexamer [112]. Interestingly, although HTLV-1 Gag is thought to localize to the PM as a monomer, previous studies have shown that HTLV-1 Gag forms disulfide-linked dimers via Cys^61^, located in the MA domain of Gag [113]. However, the biological relevance of this dimerization remains unclear. Given that HTLV-1 Gag does not seem to form dimers until it reaches the PM and that HTLV-1 Gag puncta primarily recruit additional Gag molecules from the PM rather than from the cytoplasm, it is plausible that HTLV-1 and HIV-1 assemble in a broadly similar manner but through different mechanisms.

## 10. Gag Targeting to the Plasma Membrane

During the late phase of the infection cycle, retroviral Gag polyproteins are targeted to the PM for assembly, budding, and virus release [108,114,115,116,117,118,119,120,121,122,123,124,125]. For most retroviruses, including HTLV-1, Gag proteins undergo post-translational modification in which a myristoyl (myr) group is added to the N-terminus of the MA domain of Gag. Gag binding to the PM is mediated by the MA domain, which for most retroviruses contains a bipartite signal consisting of the myr group and a highly basic region (HBR). The HBR is a conserved feature in MA proteins across multiple genera within the *Retroviridae* family [121,126,127]. Studies have established that the assembly of Gag at the PM is influenced by several factors such as protein multimerization, the presence of cellular and viral RNA, and the type of lipids as well as the saturation levels of acyl chains [119,120,128,129,130,131,132,133,134,135,136,137,138,139,140,141,142,143,144,145,146].

For many retroviruses including HIV-1, Gag targeting to the PM has been shown to be dependent on phosphatidylinositol 4,5-bisphosphate (PI(4,5)P_2_) [123,125,144,147,148], a signaling lipid in the inner leaflet of the PM [149]. Overexpression of polyphosphoinositide 5-phosphatase IV (5ptaseIV), which cleaves the 5′-phosphate group from PI(4,5)P_2_, thus depleting it within the cell, resulted in a marked decrease in HIV-1 Gag localization to the PM and hence virus-like particles (VLPs) [120]. In addition to HIV-1, other retroviruses such as equine infectious anemia virus, murine leukemia virus (MLV), ASV, and Mason-Pfizer monkey virus showed sensitivity to PI(4,5)P_2_, implicating PI(4,5)P_2_ as a major determinant for PM targeting of retroviral Gag and virus assembly and release [120,123,143,144,147,150,151,152,153].

The inner leaflet of the PM contains approximately 1 mol% of PI(4,5)P_2_ and/or PI(3,4,5)P_2_, while negatively charged lipids like phosphatidylserine (PS) are more abundant, making up about 10 mol% [154,155]. Similar to PI(4,5)P_2_, PS is preferentially found on the inner leaflet of the PM and in endocytic membranes, potentially serving as an attractive partner for cellular proteins targeting the PM [156]. Synergistic binding between PS and PI(4,5)P_2_ has been observed for the MA protein of ASV, indicating that some retroviruses may utilize PS for membrane targeting in addition to PI(4,5)P_2_ [150], suggesting that some retroviruses may engage PS for membrane targeting in addition to PI(4,5)P_2_. However, as most studies on retroviral MA proteins have focused on specific interactions of MA with PI(4,5)P_2_, the role of PS in membrane targeting and whether MA proteins can engage PS via a specific binding site remains unclear. For HIV-1, MA interacts with PS but requires PI(4,5)P_2_ in order to induce cluster formation [157].

Compared to HIV-1, HTLV-1 Gag exhibits less sensitivity to the depletion of PI(4,5)P_2_ caused by the overexpression of 5ptaseIV, indicating that PI(4,5)P_2_ may play a lesser role in HTLV-1 replication [143]. While HIV-1 binds much more effectively to liposomes containing both PS and PI(4,5)P_2_, HTLV-1 Gag has shown similar binding to liposomes with either PS alone or PS combined with PI(4,5)P_2_, as long as the overall negative charge is preserved. These findings suggest that PI(4,5)P_2_ is not crucial for HTLV-1 Gag’s membrane binding, which is likely driven by electrostatic interactions [143]. Previous studies have shown that HIV-1 MA binding to RNA negatively regulates membrane binding by preventing the HBR from interacting with membranes that lack PI(4,5)P_2_, thus preventing non-specific binding [128,135,158]. Comparative studies of HTLV-1 and HIV-1 Gag binding revealed that treating HIV-1 Gag-like constructs with RNAse increased their affinity for negatively charged membranes, whereas the interaction of HTLV-1 MA with membranes remained unaffected by RNAse treatment. This suggests that RNA interactions are less significant for HTLV-1 than for HIV-1 [143,144].

## 11. How HIV-1 and HTLV-1 MA Interact with Lipids and Membranes

**Figure 3:**
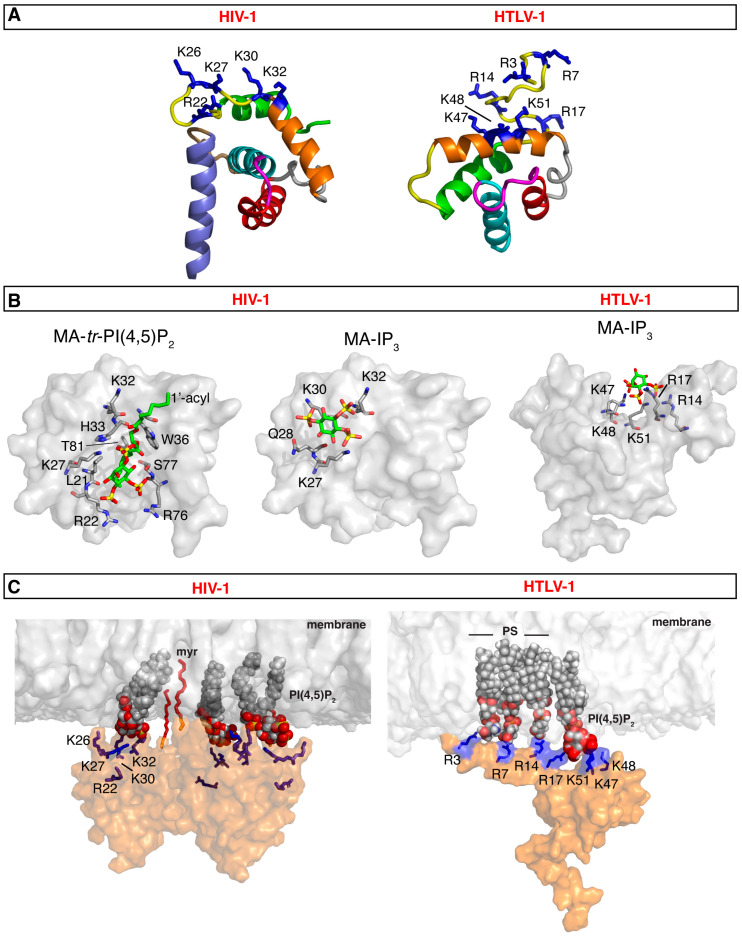
MA-membrane binding models for HIV-1 and HTLV-1. (**A**) Structures of HIV-1 myrMA (PDB code 2H3I) and HTLV-1 myr(–)MA (PDB code 7M1W). Structures highlight the HBR implicated in membrane binding (blue sticks). For HIV-1 myrMA, the following residues are not shown for clarity: myr group, residues 2–3 and 115–132. For HTLV-1 myr(–)MA, the following residues are not shown for clarity: 1–2 and 94–99. (**B**) Surface representation of the HIV-1 myrMA structure (PDB code 2H3I) highlighting residues that exhibited substantial chemical shift changes upon binding of *tr*-P(4,5)P_2_ (PDB code 2H3V) and IP_3_ (left and middle, respectively). Structures are viewed in identical orientations. The structure of HTLV-1 myr(–)MA bound to IP_3_ is shown on the right. (**C**) Models of HIV-1 myrMA and HTLV-1 myr(–)MA bound to membrane showing interactions between PI(4,5)P_2_ and/or PS and the HBR. Membrane bilayer was constructed by CHARMM-GUI [169].

The MA domain of Gag is a relatively small α-helical protein, consisting of four to five α-helices, with an unstructured C-terminus that serves as a flexible linker to the CA domain (Figure 3A). Ray crystallography studies of HIV-1 unmyristoylated MA [myr(–)MA] revealed that the protein adopts a trimer arrangement [159]. In contrast, NMR studies indicate that myr(–)MA exists as a monomer in solution [160,161,162,163,164,165]. Structural and biophysical studies of the HIV-1 myristoylated MA (myrMA) protein have shown that it exists in a monomer–trimer equilibrium with the myr group adopting both sequestered and exposed conformations, leading to the proposed myr switch mechanism [162,163,164]. The myr switch can be modulated by factors such as membrane association, inclusion of the CA domain, and solution pH [162,163,165]. The MA–MA interface within the trimer has been characterized using hydrogen-deuterium exchange mass spectrometry, aligning with the proposed interface observed in the crystal structure of the HIV-1 myr(–)MA trimer [166]. The trimer interface was consistent with the proposed MA–MA interface observed in the crystal structure of the HIV-1 myr(–)MA trimer [166]. Additionally, studies suggest that HIV-1 myrMA can form higher-order oligomers, such as hexamers of trimers, on membranes containing PI(4,5)P_2_ [139,167]. In fact, the X-ray structure of myrMA indicates that it can form a hexameric lattice even in the absence of a membrane [168]. The trimer–trimer interactions in the lattice are mediated by the N-terminal loop of one MA molecule and α-helices I-II, and the 3_10_ helix of an MA molecule from an adjacent trimer.

Pioneering NMR-based structural studies have shown that HIV-1 myrMA binds directly to PI(4,5)P_2_ analogs with truncated acyl chains (*tr*-PI(4,5)P_2_), inducing a conformational change that triggers myr exposure [163]. It was also found that *tr*-PI(4,5)P_2_ adopts an “extended lipid” conformation in which the 2′-acyl acid chain and the inositol head group bind to a hydrophobic cleft, while the 1′-acyl acid and exposed myr group bracket the HBR (Arg^22^, Lys^26^, Lys^27^, Lys^30^, and Lys^32^) [163]. In addition to the HBR residues, Trp^36^, Arg^76^, Thr^81^, and Ser^77^ were implicated in the binding of *tr*-PI(4,5)P_2_ (Figure 3B). Recent cryo-ET data suggested that PI(4,5)P_2_ may bind to myrMA differently in the immature vs. mature HIV-1 particles [170]. This has prompted investigations into an alternate binding mechanism for PI(4,5)P_2_ binding mechanism [163]. NMR studies of myrMA binding to inositol 1,4,5-trisphosphate (IP_3_), the polar headgroup of PI(4,5)P_2_, revealed that Lys^27^, Gln^28^, Lys^30^, and Lys^32^ constitute the IP_3_ binding site. These residues, located in the HBR, do not reside within the hydrophobic cleft (Figure 3B) [163]. NMR and cryo-ET studies suggested that PI(4,5)P_2_ binds to HIV-1 myrMA through two distinct structural sites during the processes of assembly and maturation [168,170]. In a recent study, computational approaches utilizing long-timescale molecular dynamics simulations of the myrMA multimeric assemblies of immature and mature virus particles using a realistic asymmetric membrane model focused on the myrMA-lipid interactions and the lateral organization of lipids around myrMA complexes [171]. It was shown that the mature myrMA complex exhibits a greater number of stable interactions with PS and PI(4,5)P_2_ at the trimeric interface compared to the immature complex. Notably, an alternative PI(4,5)P_2_-binding site in the immature myrMA complex was identified, where the PI(4,5)P_2_ headgroup interacted with multiple basic amino acids, including Arg^4^, Lys^30^, and Lys^32^. It was also shown that in contrast to the immature myrMA complex, the 2′-acyl chains of two PI(4,5)P_2_ molecules in the mature myrMA complex occupied binding pockets formed by residues in helix-4, as previously reported in the NMR study [163]. Together, these findings support a mechanism by which HIV-1 MA binding to the PM is mediated by the myr group, the HBR and possibly the acyl chains of PI(4,5)P_2_ (Figure 3C).

Recent NMR studies of HTLV-1 myr(–)MA revealed that the globular domain, spanning residues 21 to 93, comprises four α-helices, while residues 94 to 130 exhibit conformational flexibility (Figure 3A) [172]. Similar to HIV-1, HTLV-1 myr(–)MA exists as a monomer in solution. Currently, there is no structural data available for the HTLV-1 myrMA protein, and it remains unclear whether the myr group adopts sequestered or exposed conformations as observed for HIV-1. Previous work has indicated that the myr group is essential for membrane targeting of HTLV-1 Gag in cells [113,173,174]. However, unlike the typical observations with HIV-1, the binding of the HTLV-1 Gag G2A mutant, which lacks the myr group, is not entirely inhibited when expressed in cells, as shown by dual-color z-scan fluorescence fluctuation spectroscopy [107]. Overall, HTLV-1 MA–membrane interactions appear to be more robust than for HIV-1, likely due to stronger affinities for anionic phospholipid membranes and the absence of MA–RNA interactions that could hinder membrane binding. The potential independence from PI(4,5)P_2_ for Gag–PM binding suggests an alternative binding mechanism, allowing MA to compensate for the lack of PI(4,5)P_2_ specificity, possibly by interacting more effectively with other membrane lipids, such as PS.

Structural, biophysical, and biochemical studies revealed that HTLV-1 myr(–)MA contains a HBR harboring a PI(4,5)P_2_ binding site (HBR residues: Arg^3^, Arg^7^, Arg^14^, Arg^17^, Lys^47^, Lys^48^, and Lys^51^) [172,175]. Despite the lack of PI(4,5)P_2_ dependence in cells, soluble analogs of PI(4,5)P_2_ bind with significantly higher affinity in vitro compared to other retroviral MA proteins that do depend on PI(4,5)P_2_ for assembly, including HIV-1 [172]. Specifically, the affinity of HTLV-1 myr(–)MA to soluble analogs of PI(4,5)P_2_ was >20-fold higher than that observed for HIV-1, and ~100-fold higher than for ASV MA [172]. However, when evaluating lipid interactions in the context of membranes, myr(–)MA demonstrated about three-fold lower affinity for PI(4,5)P_2_ compared to HIV-1 myrMA [161,172]. The presence of PS enhanced the binding affinity of HTLV-1 myr(–)MA to PI(4,5)P_2_, suggesting a synergistic effect. The incorporation of PS in large unilamellar vesicles (LUVs) yielded a similar fraction of protein-bound as in PI(4,5)P_2_–enriched LUVs if the total negative charge was maintained, indicating a charge-based rather than a lipid-specific binding mode [172]. For HIV-1, it was demonstrated that liposomes containing both PS and cholesterol bound myrMA with significantly greater affinity than those with only PS or cholesterol [161], supporting proposals that cholesterol may indirectly enhance the ability of PS to bind MA [133]. Altogether, structural studies confirmed that HTLV-1 MA contains a PI(4,5)P_2_ binding site with binding primarily driven by electrostatic interactions rather than specific interactions with PI(4,5)P_2_. Consistent with this observation, HTLV-1 myr(–)MA lacked specificity to the location of the phosphate group as PI(4,5)P_2_ and PI(3,5)P_2_ bound with a similar affinity [172]. This result is analogous to that observed for HIV-1 MA [161]. Given that PI(3,5)P_2_ is approximately 100-fold less abundant in cells than PI(4,5)P_2_ [176], efficient Gag binding to the PM is likely a result of the higher relative concentration of PI(4,5)P_2_ rather than differences in MA affinity [161].

In a follow-up study, it was found that the PI(4,5)P_2_ binding site in HTLV-1 MA features a lysine-rich motif comprised of Lys^47^, Lys^48^, and Lys^51^ (Figure 3B). Substituting all three lysine residues significantly impaired binding to both IP_3_ and LUVs containing PI(4,5)P_2_ [175]. Additionally, an arginine-rich motif (Arg^3^, Arg^7^, Arg^14^, and Arg^17^) was identified as essential for MA binding to membranes containing PS and/or PI(4,5)P_2_ (Figure 3B) [175]. The disruption of the PI(4,5)P_2_ binding site by substitution of the three lysine residues abolished binding to LUVs containing PI(4,5)P_2_. Equilibrium flotation centrifugation and fluorescence z-scan analyses further demonstrated the importance of the lysine-rich motif in membrane targeting of Gag [175]. Interestingly, the HTLV-1 myr(–)MA triple-lysine mutant bound to LUVs containing PS with similar efficiency as wild-type (wt), indicating that the interaction with PS is facilitated by the arginine-rich motif [175]. The substitution of the Lys- and/or Arg-rich regions severely attenuated VLP production, indicating that these sites are critical for virus assembly [175]. Collectively, these findings support a mechanism by which HTLV-1 MA binding to the PM is mediated by the myr group, structured Lys-rich, and unstructured Arg-rich motifs (Figure 3C). In summary, data support a novel mechanism by which HTLV-1 Gag targeting the PM is mediated by the myr group and the Arg- and Lys-rich motifs, governed by charge-charge interactions, and is enhanced by acidic lipids such as PI(4,5)P_2_ and PS. These findings emphasize key differences in the assembly pathways of HTLV-1 compared to other retroviruses, including HIV-1.

## 12. Capsid-Capsid Interactions

**Figure 4:**
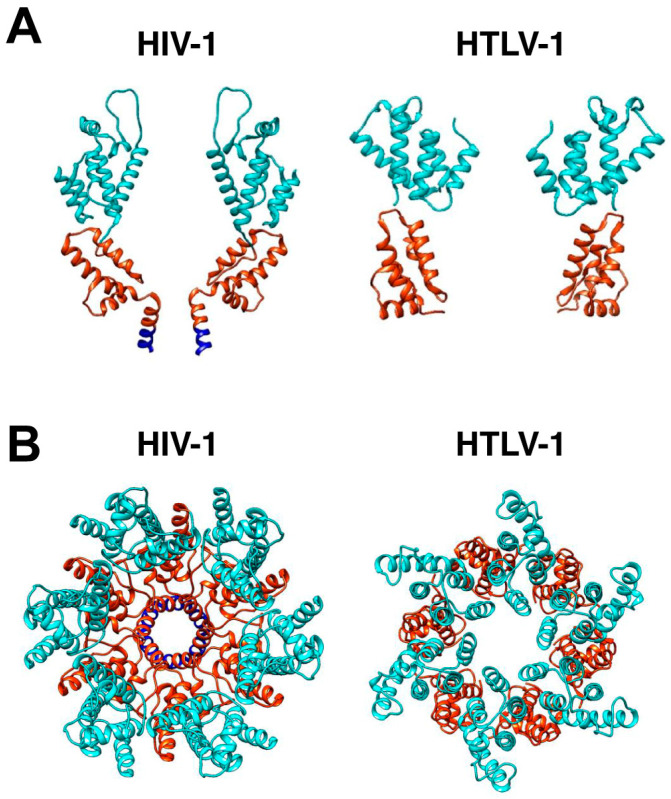
HIV-1 and HTLV-1 Gag hexamer structures. (**A**) The two HIV-1 CA molecules are displayed on the side of the hexamer, with CA_NTD_ in cyan and CA_CTD_ in orange. HIV-1 SP1 domains are shown in blue. The PDB codes are HIV-1 (5L93) [109], HTLV-1 CA_NTD_ (8PUG) [183], and HTLV-1 CA_CTD_ (8PUH) [183]. The cross-section of the HTLV-1 Gag lattice reconstruction map suggests a distinctive arrangement of the CA_NTD_ and CA_CTD_ compared to HIV-1. (**B**) Shown is the top view of the HIV-1 hexamer structure, which was generated by fitting HIV-1 CA (5L93) into the EM density of the immature HIV-1 lattice (EMD: 4017). The top view of the HTLV-1 Gag hexamer structure shown was generated by fitting CA_NTD_ and CA_CTD_ separately into the EM density of the immature HTLV-1 CA lattice (EMD: 17942). The flexible linker between HTLV-1 CA_NTD_ and CA_CTD_ is unstructured and is therefore not shown.

Following HIV-1 Gag proteolysis, CA spontaneously assembles into a fullerene cone housing the genome, viral enzymes (IN and RT), and some accessory proteins. This CA core consists of approximately 1500 CA monomers assembled into 250 hexamers and exactly 12 pentamers to facilitate the curvature on the top and bottom of the core necessary to form a closed structure [177]. The CA protein consists of two independently folded subdomains, the N-terminal domain (CA_NTD_) and the C-terminal domain (CA_CTD_) (Figure 4A). For HIV-1, mutations in the CA_CTD_ were shown to severely impair viral infectivity, number of virions, and cone formation [101]. Inositol hexaphosphate (IP_6_) was recently identified as an essential cofactor for CA assembly of HIV-1 and ASV [178,179]. In both HIV-1 and ASV, IP_6_ is localized within the core of the CA hexamer, coordinated via two rings of positively charged residues (Arg^18^ and Lys^25^), thus stabilizing the hexamer structure. IP_6_ has been shown to dramatically enhance immature particle assembly; the depletion of IP_6_ from cells or the mutation of residues that bind IP_6_ led to severely attenuated particle production and infectivity [180,181]. It has also been shown that an immature HIV-1 Gag lattice is required to concentrate IP_6_ into virions to catalyze mature CA assembly [182]. Disabling the ability of HIV-1 to enrich IP_6_ does not prevent immature Gag lattice formation or production of the virus. However, without sufficient IP_6_ molecules in each virion, HIV-1 can no longer build a stable CA and fails to become infectious [182].

The cryo-ET and subtomogram averaging of HTLV-1 immature particles have further characterized the novel HTLV-1 immature Gag lattice stabilization that is driven by the CA_NTD_ [183]. Comparative analysis of the side views of the HIV-1 and HTLV-1 Gag hexamer structures provides evidence that the cross-section of the HTLV-1 Gag lattice reconstruction map forms a distinctive arrangement of the CA_NTD_ and CA_CTD_ compared to that of HIV-1 (Figure 4B), supporting the distinct structural differences between these viruses.

For HTLV-1, previous studies have estimated that, on average, approximately 1300 to 1600 copies of Gag are packaged in HTLV-1 immature particles [184]. In contrast to HIV-1, the HTLV-1 immature CA core has an unordered polyhedral-like structure that can vary in size. The HTLV-1 immature Gag lattice has regions with a curvature that follows the viral membrane, and other regions that have a flattened lattice morphology that can be distant from the viral membrane. The HTLV-1 CA_NTD_ consists of a β-hairpin and a centralized coiled-coil-like structure of six α-helices, and a CA_CTD_ that contains four α-helices that are connected by a flexible linker (Figure 4A). Previous utilization of a panel of Gag proteins with chimeric HIV-1/HTLV-1 CA domains helps to identify distinct differences between the HIV-1 and HTLV-1 CA_NTD_ and CA_CTD_ [185]. In particular, the Gag protein expressing a CA chimera with HIV-1 CA_NTD_ and HTLV-1 CA_CTD_ did not result in Gag oligomerization or virus particle release regardless of the parental Gag background. Without CA-driven dimerization, the chimeric-CA Gag proteins in the HTLV-1 background could be translocated to the PM; in contrast, in the HIV-1 background, the chimeric-CA Gag proteins remained largely in the cytoplasm [185]. This observation, along with the observation that chimeric Gag proteins with the HTLV-1 CA_NTD_ produced particles that were morphologically similar to those of HTLV-1 particles provided evidence that HTLV-1 CA_NTD_ plays a critically important role in HTLV-1-immature particle morphology. The observations that HTLV-1 CA_NTD_ can functionally replace HIV-1 CA_CTD_, but that the HIV-1 CA_NTD_ cannot replace HTLV-1 CA_CTD_ helped to establish clear differences in CA structure and function of HTLV-1 CA to that of HIV-1 CA.

Site-directed mutagenesis studies of HTLV-1 CA_NTD_ were conducted to demonstrate that the CA_NTD_ is critical for mediating Gag–Gag interactions [185,186]. Several residues (i.e., Met^17^, Gln^47^, Phe^48^, and Tyr^61^) were identified as essential for CA-CA and Gag-Gag interactions. Modeling studies suggested that Met^17^ and Tyr^61^ are located at the dimer interface, while Gln^47^ and Phe^48^ are found at the trimer interface. The novel roles of the HTLV-1 CA_NTD_ and CA_CTD_ in immature particle formation are further supported by structural studies that indicate the role of the CA_NTD_ in HTLV-1 immature Gag lattice stabilization, as well as by mutational studies of the conserved HTLV-1 major homology region in the CA_CTD_ that implicate a structural role in facilitating CA-CA interactions mediated by the CA_NTD_. Due to these distinct differences in CA-CA interactions compared to HIV-1, the HTLV-1 immature Gag lattice has a different morphology than that of HIV-1 (Figure 4B).

## 13. Env Incorporation into Viral Particles

**Figure 5:**
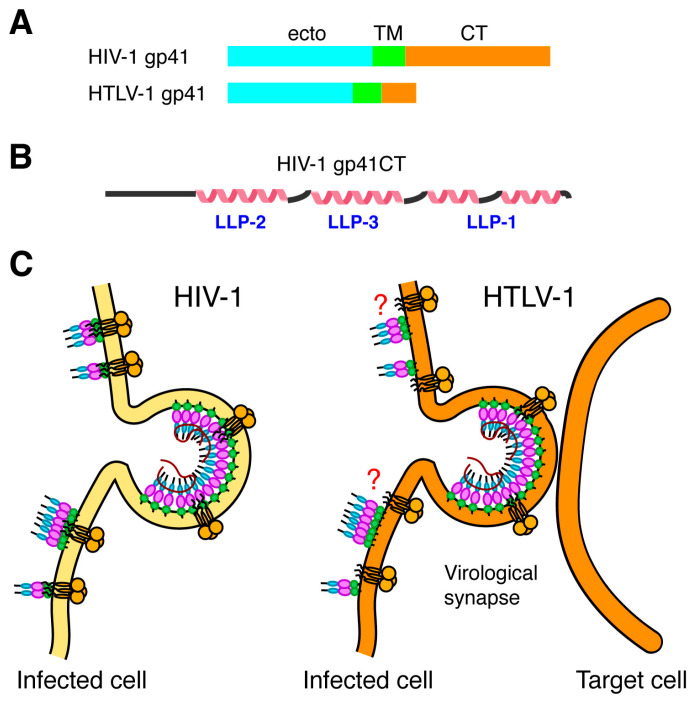
Comparison of Env CT. (**A**) Schematic representation of the gp41 subunits, indicating the lengths of their respective cytoplasmic tails (25 and 150 amino acids for HTLV-1 and HIV-1, respectively. (**B**) Secondary structure representation of the HIV-1 gp41CT protein based on the NMR data [192]. (**C**) HIV-1 Env incorporation is mediated by interaction between the MA domain of the Gag lattice and gp41CT. For HTLV-1, the CT appears to contain functional motifs that play important roles in cell-to-cell infection and syncytium formation.

For HIV-1, Gag and Env proteins are transported to the PM through independent mechanisms (Figure 1) [187,188,189,190]. Env is synthesized as a 160-kDa precursor in the rough endoplasmic reticulum, where it is glycosylated and subsequently cleaved in the Golgi apparatus to form the surface (gp120) and transmembrane (gp41) subunits (reviewed in [191]). The gp41 subunit consists of a fusogenic ectodomain, a transmembrane (TM) domain, and a C-terminal cytoplasmic tail (gp41CT) (Figure 5A). Without gp41, there is no fusion and no infectivity. Notably, the gp41CT domain is remarkably long (150 residues) for most lentiviruses but significantly shorter (20–40 residues) for other retroviruses such as HTLV-1 (Figure 5A) [191]. The biological implications of the variable length and its effect on Env incorporation are not well understood. Structural studies of gp41CT associated with detergent micelles have shown that the N-terminal 45 residues of gp41CT are disordered and do not interact with the membrane [192]. However, the C-terminal domain (residues 46–150) consists of three consecutive amphipathic α-helices (LLP2, LLP3, and LLP1) and is tightly associated with the membrane (Figure 5B) [192]. Other NMR-based studies of the TM–gp41CT domain in bicelles have shown similar structural arrangements for the gp41CT motif [193].

The mechanism by which the Env protein is recruited and incorporated into virus particles remains poorly understood. Genetic studies indicate that for HIV-1, both the gp41CT and a well-formed MA lattice (Figure 5C) are essential for incorporation and infectivity in physiologically relevant cell types [194,195,196]. It appears that it is not sufficient to only embed gp41CT in the MA layer, but it is necessary for the MA layer to undergo a cleavage-induced maturation step for gp41 to become fully active [197]. Studies have shown that the substitution of several residues in MA (L13E, E17K, L31E, V35E, and E99V) impaired Env incorporation in HIV-1 particles [189,190,198,199,200]. Notably, the substitution of residue Gln^63^ with Arg suppressed Env incorporation defects caused by the L13E, E17K, L31E, V35E, and E99V mutations, as well as a gp41CT mutation that has the same phenotype [189,190,198,199]. The Freed laboratory provided biochemical evidence that MA trimerization is an obligatory step for Env incorporation and demonstrated a correlation between loss of MA trimerization and loss of Env incorporation [199]. Structural and biophysical studies indicated that A45E, T70R, and L75G mutations in myrMA did not alter the overall structure and folding of MA and caused only minor structural perturbations in the trimer interface and had a minimal effect on the MA monomer–trimer equilibrium [201]. The X-ray structure of the myr(–)MA Q63R protein revealed hydrogen bonding between the side chains of Arg^63^ and Ser^67^, providing evidence for an additional intermolecular interaction in the trimer interface [201]. These findings provided further evidence for an interplay of MA trimerization and Env incorporation into HIV-1 particles.

It has also been reported that Gag assembly promotes the aggregation of small Env clusters into larger domains that were completely immobile; truncation of gp41CT abrogated Gag’s ability to induce Env clustering and restored Env mobility at assembly sites [202]. Super-resolution microscopy data also indicated that recruitment of Env to viral assembly sites is dependent on gp41CT [188]. Nanoscale single particle tracking of Env on the PM has demonstrated that Env immobilization at sites of Gag assembly requires gp41CT but does not require the curvature of the lattice [203]. Env was restricted to subviral regions within the Gag lattice, indicating that an interaction between gp41CT and MA may be responsible for Env retention in budding particles.

To explore the dynamics of Env recruitment, a recent study utilized a chemical dimerizer system to manipulate HIV-1 assembly through reversible depletion of PI(4,5)P_2_ as visualized by super-resolution and live-cell microscopy [204]. This method enabled the control and synchronization of HIV-1 assembly, as well as the monitoring of Env recruitment to individual nascent assembly sites in real-time. Tracking individual virions revealed that Gag and Env accumulate at HIV-1 assembly sites with similar kinetics. The depletion of PI(4,5)P_2_ hindered Gag’s targeting of the PM and prevented the formation of Env clusters, indicating that Env recruitment depends on Gag assembly. In cells with pre-assembled Gag lattices, PI(4,5)P_2_ depletion led to the disintegration of the entire assembly domain, causing the rapid loss of both Gag and Env clusters from the PM. These findings suggested that Gag induces and maintains a membrane microenvironment that attracts Env. Disruption of this microenvironment by PI(4,5)P_2_ depletion appears to result in the loss of Env from the assembly domain [204].

HTLV-1 Env is synthesized as a precursor protein (gp62), which is then folded, oligomerized, and glycogenized in the epithelial system. This precursor is then transported via the Golgi and cleaved by cell enzymes to form the surface glycoprotein (SU; gp46) and the transmembrane glycoprotein (TM; gp21) [205]. The subunits are divided into trimers, which are maintained through non-coagulation, where SU resides in the extracellular space and TM is embedded into the cell membrane or viral envelope [205]. SU and TM work together to allow viral entry. SU binds directly to cell surface receptors, whereas TM allows the fusion of viral and cell membranes. While no structural data is available on the mature SU gp46, the X-ray structure of the ectodomain of gp21 revealed a coil-coil arrangement [206]. HTLV-1 contains a short CT (24 amino acids) located at the C-terminus of TM (Figure 5A) [207]. Although structural data of the TM and CT of HTLV-1 are lacking; the CT appears to contain functional motifs that play important roles in cell-to-cell infection and syncytium formation (Figure 5C) [208,209]. Limited mutagenesis studies on HTLV-1 MA have shown Env proteins were incorporated with the mutated MA constructs at a level similar to that of the wt provirus [210]. Detailed investigation of the mechanism of Env incorporation into HTLV-1 particles is warranted

## 14. Virus Maturation

**Figure 6:**
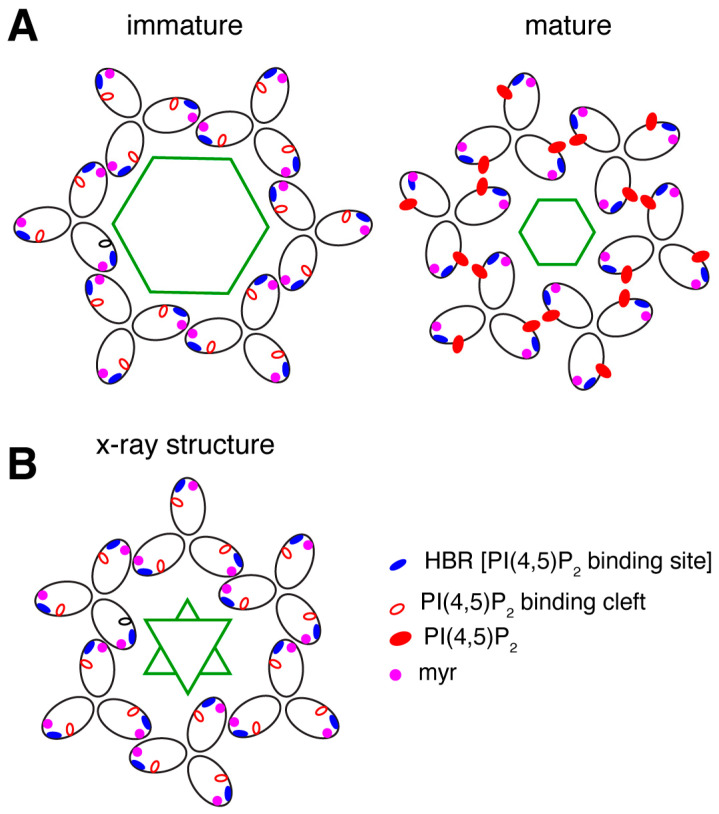
Comparison of MA lattices based on structural data. (**A**) Schematic representation of the myrMA lattice in the immature and mature states based on the cryo-ET data [170]. The trimer–trimer interactions are mediated by the N-terminal domain in the vicinity of the myr group, while the PI(4,5)P_2_ binding pocket is empty. In the mature myrMA lattice, PI(4,5)P_2_ is bound to the cleft and myrMA trimer–trimer interactions are formed by the HBR and PI(4,5)P_2_. (**B**) Schematic illustration of the myrMA lattice based on the X-ray structure of myrMA. In this lattice, myrMA–myrMA interaction at the trimer–trimer interface is mediated by the N-terminal residues. Of note, myrMA–myrMA interaction at the trimer–trimer interface places the myr groups (red) in juxtaposition. The HBR and PI(4,5)P_2_ binding cleft are also shown. Hexagons and triangles denote C6 and C3 symmetry, respectively.

Gag and Gag-Pol are incorporated into the budding virus particle at a specific ratio (20:1 Gag:Gag-Pol), a conserved feature across retroviruses. Maintaining this ratio is essential for virus structure and infectivity [95,211]. Proteolytic cleavage of Gag and Gag-Pol via the viral protease (Pro) is initiated either during or after budding, marking the final process of the retroviral life cycle: maturation. Cleavage of Gag and Gag-Pol proceeds at different rates, likely influenced by protein sequence and structure. It has been shown that, in vitro, cleavage of HIV-1 Gag occurs fastest at the SP1-NC cleavage site, followed by SP2-p6, MA-CA, NC-SP2, and, finally, CA-SP1 [212]. Cleavage initiates a cascade of major structural rearrangements to form the mature virus particle [213]. For HIV-1, changes can be summarized as follows: (1) The cleaved NC protein nucleates and condensates with the viral RNA [214,215]. (2) Cleavage of the SP1 domain and the MA domain from CA leads to a major structural rearrangement of CA which temporarily liberates IP_6_ from the hexamer and allows for the formation of the distinct viral cone shape. Within this mature CA hexamer, IP_6_ is bound to Arg^18^ located in CA_NTD_ and stabilizes the CA shell [216,217,218,219]. (3) MA remains associated with the membrane but undergoes major structural re-organization [170]. As discussed above, cryo-ET data indicated that the MA domain undergoes dramatic conformational change to allow for the formation of distinct hexameric lattices in the immature and mature particles (Figure 6) [170]. The propensity of MA to form a lattice has been reported by Barklis et al. using cryo-electron diffraction of 2D crystals of MA on a lipid monolayer [167]. As discussed above, myrMA is capable of forming a hexamer of trimers lattice even in the absence of PI(4,5)P_2_ and membrane (Figure 6) [168]. The MA lattice is increasingly seen as central to the mechanism of Env incorporation [189,190,199,220]. HTLV-1 maturation, on the other hand, is not well understood. While the proteolytic sites are known, cryo-ET studies of immature and mature virus particles of HTLV-1 have failed to reveal distinct viral lattices, even for the relatively large CA domain [184,221,222]. Furthermore, it is not known if HTLV-1 MA forms an ordered lattice in the immature or mature particles. Difficulties in obtaining structural data of HTLV-1 particles via EM are compounded by the low particle production and pleomorphic, incomplete shells rather than distinct cone-shaped structures.

## 15. Conclusions

HTLV-1 continues to be a growing and persistent threat due to its severe health impacts and the challenges associated with current treatments. Although significant progress has been made in understanding the molecular mechanisms of retroviral replication, there are still major gaps in our knowledge. While the general replication cycle is similar among retroviruses, comparative studies revealed important differences in the replication pathways and the structure and function of viral components. This review highlighted both the similarities and differences in the replication processes of HIV-1 and HTLV-1. We also discussed recent advances in understanding the molecular determinants of HTLV-1 and HIV-1 assembly, with a particular focus on the interactions between Gag and MA with the membrane, as well as CA assembly. Despite these advances, crucial aspects of HTLV-1 replication, such as virus entry, uncoating, reverse transcription, assembly, and budding, remain poorly understood.

Treatment options for HTLV-1-related conditions are currently limited, with existing therapies often being costly and not always effective. This underscores the urgent need for further research to develop more affordable and effective treatments. The progress seen in HIV-1 drug development offers hope that similar breakthroughs could lead to effective and affordable therapies for HTLV-1. The success of new HIV-1 drugs that target CA assembly, such as Lenacapavir, ref. [223] offers a promising model for HTLV-1. Insights gained from virus assembly and replication mechanisms of HIV-1 could guide the creation of analogous strategies for HTLV-1. By leveraging innovative drug development approaches, we might achieve more cost-effective treatments and potentially revolutionize HTLV-1 management. Continued research and heightened awareness are crucial for reducing the impact of this virus and improving global health outcomes.

## Data Availability

Not applicable.
